# The Measle of the Pig
†On the Measle of the Pig, and on the Wholesomeness as Food for Man of Measly Pork. By Alexander Fleming, M.D. Dublin: McGlashan and Gill. 1857.


**Published:** 1857-04

**Authors:** 


### THE MEASLE OF THE PIG.†

Dr. Fleming's labours were requested by the Committee of the Provision Merchants of Cork. Some of the questions submitted by the Committee we subjoin, with the answers in brief.

"What is the nature and origin of measle in the pig? The measle is an animal parasite, the cysticercus cellulosæ, or bladder flesh-worm.—Are all pigs measly? All pork is not measly.—Is there any analogy between measles in the pig, and the disease known by that name in man? None.—Is fresh measly pork wholesome? Is cured measly pork wholesome? We cannot

---

† On the Measle of the Pig, and on the Wholesomeness as Food for Man of Measly Pork. By ALEXANDER FLEMING, M.D. Dublin: McGlashan and Gill. 1857.

regard bad measly pork, fresh or cured, as wholesome food for man. We believe that the life of the parasite is destroyed in the process of curing.—Can pork be measly, and that condition be invisible to the naked eye? In the specimens of measled pork, the worms were all visible to the naked eye; in none were there any traces of the eggs of the parasite, or of its development in an earlier stage."

Dr. Fleming, following the modern view on the transmission of the entozoa, believes that the measle of pigs originates in the eggs of the tape-worm which infests the bowels of the dog. These, when voided by the dog, are resolved into fine dust, and are scattered by the wind, and thus, mixing with the food of the pig, enter its body. The report is ably done.

